# Reporting characteristics of journal infographics: a cross-sectional study

**DOI:** 10.1186/s12909-022-03404-9

**Published:** 2022-04-27

**Authors:** Giovanni E. Ferreira, Mark R. Elkins, Caitlin Jones, Mary O’Keeffe, Aidan G. Cashin, Rosa E. Becerra, Andrew R. Gamble, Joshua R. Zadro

**Affiliations:** 1 Institute for Musculoskeletal Health, The University of Sydney and Sydney Local Health District, Sydney, Australia; 2 School of Public Health, Faculty of Medicine and Health, The University of Sydney, Missenden Road, Camperdown, PO Box M179, Sydney, NSWNew South Wales 2050 Australia; 3 Faculty of Medicine & Health, The University of Sydney, Sydney, Australia; 4 Centre for Pain IMPACT, Neuroscience Research Australia (NeuRA), Sydney, Australia

**Keywords:** Medical education, Infographics, Information Dissemination, Visual abstracts

## Abstract

**Background:**

Infographics have become an increasingly popular method to present research findings and increase the attention research receives. As many scientific journals now use infographics to boost the visibility and uptake of the research they publish, infographics have become an important tool for medical education. It is unknown whether such infographics convey the key characteristics that are needed to make useful interpretations of the data such as an adequate description of the study population, interventions, comparators and outcomes; methodological limitations; and numerical estimates of benefits and harms. This study described whether infographics published in peer-reviewed health and medical research journals contain key characteristics that are needed to make useful interpretations of clinical research.

**Methods:**

In this cross-sectional study, we identified peer-reviewed journals listed in the top quintile of 35 unique fields of medicine and health research listed in the Journal Citation Reports database. Two researchers screened journals for the presence of infographics. We defined an infographic as a graphical visual representation of research findings. We extracted data from a sample of two of the most recent infographics from each journal. Outcomes were the proportion of infographics that reported key characteristics such as study population, interventions, comparators and outcomes, benefits, harms, effect estimates with measures of precision, between-group differences and conflicts of interest; acknowledged risk of bias, certainty of evidence and study limitations; and based their conclusions on the study’s primary outcome.

**Results:**

We included 129 infographics from 69 journals. Most infographics described the population (81%), intervention (96%), comparator (91%) and outcomes (94%), but fewer contained enough information on the population (26%), intervention (45%), comparator (20%) and outcomes (55%) for those components of the study to be understood without referring to the main paper. Risk of bias was acknowledged in only 2% of infographics, and none of the 69 studies that had declared a conflict of interest disclosed it in the infographics.

**Conclusions:**

Most infographics do not report sufficient information to allow readers to interpret study findings, including the study characteristics, results, and sources of bias. Our results can inform initiatives to improve the quality of the information presented in infographics.

**Supplementary Information:**

The online version contains supplementary material available at 10.1186/s12909-022-03404-9.

## Introduction

‘Infographic’ is an abbreviated term for an information graphic. They generally use images and data visualisations (pie charts, bar graphs, line graphs) to foster knowledge translation through increasing attention, comprehension and recall; and are considered aesthetically appealing and useful to communicate research findings among peers, the media and the public [1, 2]. Infographics have become an increasingly popular method to present research findings to non-academic audiences and increase the attention research receives [2–4]. Many scientific journals now use infographics to boost the visibility and uptake of the research they publish [5]. This includes healthcare journals with broad coverage (e.g., New England Journal of Medicine), and those focused on a specific discipline (e.g., JAMA Oncology, British Journal of Sports Medicine).

There is limited guidance on how to appropriately report research findings within infographics. To the best of our knowledge, only one guideline has been developed to inform the design of infographics (7-item GRAPHIC guidelines) [6]. However, this guideline only provides recommendations for infographic formatting.

Infographics that summarise the results of clinical research (e.g., observational studies, randomised trials, reviews) could improve knowledge translation and increase uptake of new evidence in clinical practice. However, it is unknown whether such infographics convey the key characteristics that are needed to make useful interpretations of the data. Such characteristics include but are not limited to: an adequate description of the study population, interventions, comparators and outcomes; methodological limitations; and numerical estimates of benefits and harms.

There is yet to be a systematic assessment of the reporting of key research characteristics in infographics. The aim of this study was to describe the proportion of infographics of clinical research that:

- describe the study population, interventions, comparators and outcomes (and do so well enough for the infographic to be understood independently of the main paper);
- report the benefits and harms of an intervention, effect estimates with measures of precision, between-group differences, the relationship of the effect estimates to known thresholds of clinical importance, and clear summary statistics for dichotomous outcomes;
- acknowledge risk of bias, the certainty of evidence (if applicable), and study limitations;
- acknowledge risk of bias/certainty of evidence in their conclusion, base conclusions on the correct populations, interventions or outcomes (i.e. no issue with indirectness), and base conclusions on the primary outcome; and
- report conflicts of interest.

## Methods

### Data sources

We reported this cross-sectional study following the STROBE guidelines [7]. We defined an infographic as a graphical visual representation of research findings. We only included infographics summarising clinical research studies (i.e., observational studies, randomised and non-randomised trials, systematic reviews). There was no restriction on the population, intervention, or outcomes investigated. We did not consider infographics from in vitro or in silico studies. The search strategy comprised three steps:

Step 1: One researcher selected the 35 unique fields related to health and medical research from the Journal Citation Reports database (Additional file 1: Appendices A,B). Within each field, journals ranked in the top *quintile* based on journal impact factor using data from the 2019 journal impact factor index were selected (*n* = 597 journals).

Step 2: Two researchers from a panel of six independently checked each journal’s website (*n* = 597) for infographics. This was done by searching terms synonymous with “infographic” in the journal’s search box (e.g., “graphic abstract”, “graphical abstract”, “visual abstract”), and by manually checking articles published ahead of print and in all issues from August 2018 to October 2020. We did not consider issues designated to conference proceedings, special issues, or supplements. We also searched for special sections within the journal’s website (e.g., the BMJ visual abstract and infographic Sects [8].). If no infographics were identified with those procedures, we considered the journal not to have infographics. Due to feasibility issues, we only checked the journal’s websites. Other sources such as Twitter and Facebook were not checked. This step was conducted between September 21^st^ and October 2^nd^, 2020.

Step 3: Pairs of investigators from a panel of six independently selected the two most recently published and eligible infographics from each journal. If the pair of investigators identified different infographics, they met to discuss and reach consensus.

### Data extraction

Using a standardised data extraction form that was pilot tested prior to data extraction, two investigators independently extracted data from the infographics. Investigators were provided with instructions and examples of how to code every item of interest (Additional file 1: Appendix C). Discrepancies were resolved by discussion between the pair of investigators. This method is consistent with that recommended for high-quality Cochrane systematic reviews [9]. When an item was not relevant to the study design, it was recorded as “not applicable”. For example, in an observational study with no fixed intervention, it would not be applicable to report a description of the intervention or an estimate of the between-group difference.

### Data analysis

We summarised data from the overall sample using counts and percentages. We also reported outcomes stratified by study design – differences between We calculated differences between proportions of each analysed item stratified by study design using Pearson’s Chi-squared. We used Stata version 16.1 (StataCorp LLC, Texas, USA) for the analyses.

Wherever “not applicable” was used, that infographic was not counted in the denominator for that item. The following data were extracted:

- Study design (observational study, randomised trial, or review);
- Whether the infographic described the study population, interventions, comparators, and outcomes, and whether the description was adequate for the infographic to be understood independently from the article. We considered these descriptions to be adequate when investigators did not need to check the original study report to understand key details. For example, the population needed to include some demographic characteristics (e.g., mean age). The intervention and comparison needed to include some information on the intervention parameters (e.g., drug dose, frequency of treatment). The outcome needed to be specific about the measure used (e.g., all-cause mortality).
- Whether the infographic reported benefits and harms (e.g., adverse events), effect estimates and measures of precision, and between-group differences; and presented effect estimates in relation to known thresholds of clinical importance, and a clear summary statistic for dichotomous outcomes (e.g., proportions, relative risk (RR), number needed to treat (NTT), or charts commonly used to communicate absolute risk (e.g., icon array)) [10].
- Whether the infographic acknowledged risk of bias, the certainty of evidence (if applicable), and study limitations.
- Whether the infographic had a conclusion, acknowledged limitations/risk of bias/certainty of evidence in their conclusion, based their conclusions on the correct populations, interventions or outcomes (i.e. no issue with indirectness), and based their conclusions on the primary outcome (i.e. no ‘spin’) [11].
- Whether the infographic reported conflicts of interest.

### Patient and public involvement

Patients or the public were not involved in the design, or conduct, or reporting, or dissemination plans of our research.

## Results

### Selection and characteristics of infographics

**Fig. 1:**
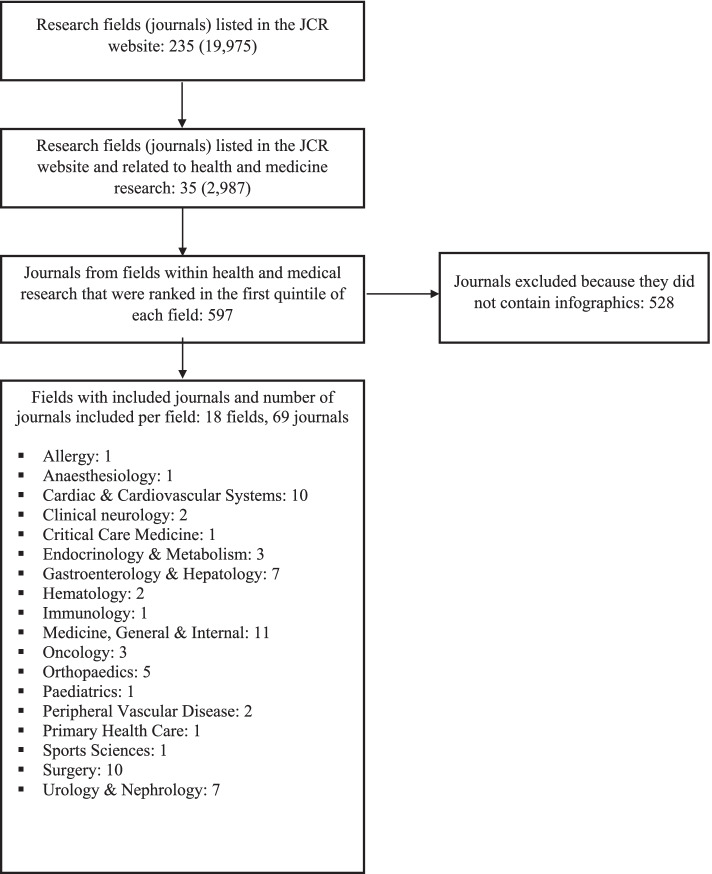
Study flow diagram

Within the 35 unique fields related to medicine and health research listed in the Journal Citation Reports database, we identified 597 journals that were listed in the top quintile of these fields. Of these, we identified 69 journals from 18 fields that contained infographics that met our eligibility criteria (Fig. 1). We were able to find two infographics from 60 journals and only one from 9 journals (Additional file 1: Appendix A). Hence, we included 129 infographics in this study.

Fields with the highest number of journals included were Medicine, General & Internal (11 journals), followed by Cardiac & Cardiovascular Systems and Surgery (10 journals each), Gastroenterology & Hepatology and Urology & Nephrology (7 journals each). The other 14 fields contributed fewer journals, ranging from 1 to 5 per field (Fig. 1). Most infographics summarised observational studies (50%), followed by randomised trials (35%) and reviews (16%) (Table 1). Of the 20 reviews included, 65% included randomised controlled trials only.

**Table 1:** Characteristics of infographics summarising studies evaluating the effects of an intervention (*n* = 129 unless stated otherwise). *P*-values are for differences in proportions in each outcome stratified by study design

Characteristics	Total (*n* = 129)	Observational Study (*n* = 64)	Randomised trial (*n* = 45)	Review (*n* = 20)	*p*-value
**Population**					
Population was described	105 (81)	52 (81)	38 (84)	15 (75)	0.66
Description of population allows the infographic to be read independently	34 (26)	16 (25)	15 (33)	3 (15)	0.28
**Intervention**					
Interventions were described	124 (96)	60 (94)	45 (100)	19 (95)	0.24
Description of interventions allows the infographic to be read independently	58 (45)	29 (45)	23 (51)	6 (30)	0.28
**Comparator** *(n* = *109 had a comparator)*					
Comparators were described	99 (91)	40 (91)	45 (100)	14 (70)	0.001*
Description of comparators allows the infographic to be read independently	55 (50)	20 (45)	28 (62)	7 (35)	0.09
**Outcomes**					
Outcomes were described	121 (94)	61 (95)	45 (100)	15 (75)	< 0.001*
Description of outcomes allows the infographic to be read independently	71 (55)	37 (58)	26 (58)	8 (40)	0.33
**Benefits & harms**					
Benefits were reported	109 (84)	50 (78)	43 (96)	16 (80)	0.03*
Harms were reported (e.g., adverse events)	33 (26)	11 (17)	12 (27)	10 (50)	0.01*
**Results**					
Effect estimates reported	87 (67)	48 (75)	32 (71)	7 (35)	0.003*
Measures of imprecision reported	28 (22)	11 (17)	14 (31)	3 (15)	0.16
Between-group differences *(n* = *109 had a comparator)*	63 (58)	27 (61)	29 (64)	7 (35)	0.07
Effect sizes were presented in relation to known thresholds of clinical importance	5 (4)	1 (2)	2 (4)	2 (10)	0.22
Dichotomous outcomes were clearly labelled^a^ *(n* = *97 had a dichotomous outcome)*	63 (65)	37 (65)	22 (79)	4 (33)	0.02*
**Bias**					
Risk of bias acknowledged	3 (2)	0 (0)	0 (0)	3 (15)	< 0.001*
Certainty of evidence mentioned *(n* = *20 reviews)*^b^	2 (10)	N/A	N/A	N/A	N/A
**Study limitations** acknowledged	1 (1)				
**Conclusion** *(n* = *63 had a conclusion)*					
Conclusions were presented considering risk of bias	3 (5)	1 (4)	0 (0)	2 (22)	0.02*
Conclusion had no issues with indirectness^c^	58 (92)	25 (93)	25 (93)	8 (89)	0.93
Conclusions were based on findings from the primary outcome	54 (86)	24 (89)	23 (85)	7 (78)	0.70
**Conflict of interest** *(n* = *69 studies declared a conflict of interest)*					
Infographic reports conflicts of interest	0 (0)	0 (0)	0 (0)	0 (0)	N/A^d^

^a^A labelled summary statistic (e.g., proportions, relative risk) or a visual representation of the data (e.g., a Cates plot) was presented

^b^Stratified analysis not presented as this item is only relevant to reviews

^c^Conclusions were based on the correct populations, interventions or outcomes

^d^*p*-value could not be computed

### Main findings

Most infographics described the population (81%), intervention (96%), comparator (91%) and outcomes (94%) of the study. However, fewer infographics contained enough information on the population (26%), intervention (45%), comparator (50%) and outcomes (55%) for these components of the study to be understood without referring to the main paper.

Fewer infographics reported harms (26%) compared to benefits (84%). Only 67% and 22% reported an effect estimate and measures of imprecision around an effect estimate, respectively. Risk of bias was acknowledged in only 2% of infographics, and certainty of evidence was only mentioned by 10% of infographics of systematic reviews. Of the 63 infographics that contained a conclusion, most (92%) did not have issues with indirectness, and most were based on findings from the primary outcome (86%). Only 5% of these conclusions considered risk of bias. None of the 69 studies that declared a conflict of interest disclosed it in the infographics.

There were some differences in some of the outcomes when data were stratified by study design. These data are displayed in Table 1. A higher proportion of infographics from observational studies and randomised trials described the comparators, outcomes, effect estimates, and clearly labelled dichotomous outcomes compared to reviews. A higher proportion of randomised trials reported on the benefits of an intervention compared to observational studies and reviews, whereas a higher proportion of reviews reported harms and acknowledged risk of bias compared to observational studies and randomised trials.

## Discussion

### Summary of findings

Infographics typically presented information on patients, interventions, comparators and outcomes. However, fewer reported sufficient information to allow readers to understand them without reference to the main paper. Critical aspects of results such as reporting measures of imprecision around the effect estimate or clearly labelling the statistic used to summarise dichotomous outcomes were seldom reported. Sources of bias, certainty of evidence, and study limitations were rarely acknowledged. No infographic disclosed conflicts of interest even though more than half of the original studies in our sample had originally disclosed at least one source of conflict of interest.

### Implications

Infographics have been shown to increase measures of research attention such as engagement on social media and Altmetric scores [5, 12, 13], yet our results indicate that in many cases the increase in attention may be at odds with high quality information. This is concerning because the absence of key information in the infographic may compromise the reader’s ability to truly understand the study and its findings, limitations, and implications for clinical practice. These limitations could be addressed by reading the full text provided that the full-text was reported following best practices in reporting eg adhered to reporting guidelines. However, whilst infographics are often made freely available on social media [5] or in dedicated sections on journal websites [14], access to the original studies is often restricted by journal paywalls.

Most infographics that had a conclusion reported findings for the primary outcome of the study (86%). In other words, only 14% of infographics were considered to have some form of spin. The proportion of spin in our sample was much lower than spin in other samples (26% to 85%) depending on the study design [15]. This could be explained by more rigorous assessments of spin being used in other studies [15–17].

None of the infographics included in our study disclosed conflicts of interest, although more than half of the studies from our sample had some form of conflict of interest declared. Conflicts of interest are an important source of bias in clinical research [18], so this information should be present in every resource designed to disseminate study findings.

### How an infographic should look like

Anyone creating an infographic could consider the items that were assessed in our study and ensure that any items that are relevant to the study design being summarised are included in the infographic. Reporting checklists for infographics for individual study designs would simplify that process and our group has commenced preparing these. In proposing such reporting checklists, we acknowledge that some items may be more or less relevant depending on the purpose of the infographic, such as notifying the general public about the existence of a new study versus informing clinicians about the evidence generated by that study. Depending on the purpose and on the format in which the infographic will be distributed (e.g., social media, journal website, poster, other), all relevant items may not be incorporated in every infographic but, in general, the more items on the checklist that are incorporated in the infographic the more informative it will be.

One item that we choose to highlight here is risk of bias. This was achieved by only 2% of the infographics we analysed, but it can be succinctly summarised, as shown in the infographic for the study by van de Leemkolk et al. [19].

The infographics that satisfied more relevant items than most infographics were the ones produced by JAMA [20, 21]. Apart from providing more complete information than others, their layout makes its interpretation clear and easy. For example, they seemed to conform well to the GRAPHIC principles that are recommended for visual presentation of infographics: restricted colours, aligned elements, prioritise parts, highlight the heading, good imagery, and careful selection of charts [22]. From the sample of infographics that we analysed in our study, the ones produced by JAMA have the best combination of reporting completeness and aesthetic appeal.

Our team has recently completed a survey (submitted for publication – data not available yet) conducted with consumers of infographics summarising health or medical research (eg health professionals, researchers, academics and patients/the public) and found that 41% used infographics as a substitute for the full-text at least half of the time, 55% thought infographics should be detailed enough so they do not have to read the full text, and 64% viewed infographics as tools to reduce the time burden of reading the full text.

### Study limitations

Searching for infographics is challenging because nomenclatures vary by journal (e.g., infographic, graphical abstract, visual abstract) and journal policies are unclear about whether infographics are routinely produced for all published papers. Furthermore, there are currently thousands of medical journals, so searching for infographics across all journals is not feasible. Most infographics cannot be located by searching indexing databases like PubMed. To overcome these limitations, we designed a comprehensive multi-step search that allowed us to search a large number of journals (*n* = 597) across 35 research fields. Our definition of infographics was broad, which allowed us to capture a broad range of different types of infographics (e.g. visual abstracts, graphical abstracts, infographics). A potential limitation of this approach is that different types of infographics might have been lumped together. However it is worth mentioning that the terms currently used by different journals to describe the various types of infographics they produce is not standardised. Future research could develop a classification system for different types of infographics and repeat our analysis.

We limited searches to journals in the top quintile of each research field, which could be considered a limitation. Although such journals typically enforce use of reporting checklists in their published papers [23], the information contained in their infographics was consistently insufficient for a useful interpretation of the data. Because journals in the other quintiles typically use less robust reporting in their papers, we anticipate that their infographics would also be insufficiently detailed for readers to make a useful interpretation of the data. In any case, the implication for clinicians remains the same regardless of the journal’s impact factor: unless an infographic reports the necessary details for clinical decision making in the infographic, users should read the full-text publication.

## Conclusion

Most infographics do not report sufficient information to allow readers interpret study findings, including the study characteristics, results, and sources of bias. Our results can inform initiatives to improve the quality of the information presented in infographics. While infographics could be made more informative, clinicians and other users of clinical research should not only rely on infographics for decision-making. These decisions should only be made after reading the full-text publication.

## Supplementary Information

**Additional file 1: Appendix A.** Fields ofmedicine and health research. **Appendix B.** List ofincluded journals and number of infographics included from each journal. **Appendix C.** Coding instructions.

## Data Availability

All data generated or analysed during this study are included in this published article.
