# Supplementary material for: Reporting characteristics of journal infographics: a cross-sectional study
Source: BMC Med Educ. 2022 Apr 27;22:326. doi: 10.1186/s12909-022-03404-9 (PMC9047312; doi:10.1186/s12909-022-03404-9)
Supplement: Supplementary file 1 — Additional file 1: Appendix A. Fields ofmedicine and health research. Appendix B. List ofincluded journals and number of infographics included from each journal. Appendix C. Coding instructions. [file 12909_2022_3404_MOESM1_ESM.docx]

**Additional file 1.**

**Appendix A. Fields of medicine and health research.**

| **Research fields** | **Rank**  **(total cites)** | **No. Journals** | **No. journals within the top quintile** |
| --- | --- | --- | --- |
| Oncology | 1 | 244 | 49 |
| Medicine, General & Internal | 2 | 165 | 33 |
| Clinical Neurology | 3 | 204 | 41 |
| Immunology | 4 | 158 | 32 |
| Surgery | 5 | 210 | 42 |
| Cardiac & Cardiovascular Systems | 6 | 138 | 28 |
| Endocrinology & Metabolism | 7 | 143 | 29 |
| Psychiatry | 8 | 155 | 31 |
| Gastroenterology & Hepatology | 9 | 88 | 18 |
| Hematology | 10 | 76 | 15 |
| Peripheral vascular disease | 11 | 65 | 13 |
| Infectious diseases | 12 | 93 | 19 |
| Pediatrics | 13 | 128 | 26 |
| Orthopedics | 14 | 82 | 16 |
| Psychology | 15 | 77 | 15 |
| Respiratory System | 16 | 64 | 13 |
| Sports Sciences | 17 | 85 | 17 |
| Urology & Nephrology | 18 | 85 | 17 |
| Obstetrics & Gynecology | 19 | 82 | 16 |
| Ophthalmology | 20 | 60 | 12 |
| Pathology | 21 | 78 | 16 |
| Virology | 22 | 37 | 7 |
| Critical Care Medicine | 23 | 36 | 7 |
| Dermatology | 24 | 68 | 14 |
| Geriatircs & Gerontology | 25 | 51 | 10 |
| Rheumatology | 26 | 32 | 6 |
| Anesthesiology | 27 | 32 | 6 |
| Rehabilitation | 28 | 68 | 14 |
| Transplantation | 29 | 24 | 5 |
| Allergy | 30 | 28 | 6 |
| Substance abuse | 31 | 36 | 7 |
| Tropical Medicine | 32 | 23 | 5 |
| Women's studies | 33 | 45 | 9 |
| Primary Health Care | 34 | 19 | 4 |
| Andrology | 35 | 8 | 2 |
| **TOTAL** |  | **2,987** | **597** |

**Appendix B. List of included journals and number of infographics included from each journal.**

| **Journal** | **No. Infographics included** |
| --- | --- |
| Allergy | 2 |
| American Journal Of Gastroenterology | 2 |
| American Journal of Kidney Diseases | 2 |
| Anesthesiology | 2 |
| Annals Of Family Medicine | 1 |
| Annals Of Internal Medicine | 2 |
| Annals Of Surgical Oncology | 2 |
| Annals Of Thoracic Surgery | 2 |
| Arteriosclerosis Thrombosis and Vascular Biology | 2 |
| Arthroscopy-The Journal of Arthroscopic And Related Surgery | 2 |
| Bju International | 2 |
| Blood | 2 |
| Bone & Joint | 2 |
| Bone And Joint Research | 2 |
| British Journal of Sports Medicine | 2 |
| British Medical Journal | 2 |
| Canadian Medical Association Journal | 2 |
| Cancer Treatment Reviews | 2 |
| Cell Metabolism | 2 |
| Chest | 2 |
| Circulation Research | 2 |
| Circulation-Cardiovascular Interventions | 2 |
| Clinical Gastroenterology And Hepatology | 2 |
| Clinical Journal of The Americian Society Of Nephrology | 2 |
| Clinical Orthopaedics and Related Research | 2 |
| Clinical Research In Cardiology | 2 |
| Critical Reviews in Oncology/Hematology | 2 |
| Diabetologia | 2 |
| Diseases of The Colon & Rectum | 1 |
| Endoscopy | 2 |
| European Heart Journal | 2 |
| European Heath Journal-Cardiovascular Imaging | 1 |
| European Journal of Cardio-Thoracic Surgery | 2 |
| European Journal of Internal Medicine | 1 |
| European Journal of Vascular And Endovascular Surgery | 2 |
| European Urology | 2 |
| Free Radical Biology and Medicine | 1 |
| Gastroenterology | 2 |
| Gastrointestinal Endoscopy | 2 |
| Heart Rhythm | 2 |
| Hypertension | 2 |
| International Journal Of Surgery | 2 |
| JACC-Cardiovascular Imaging | 2 |
| JACC-Cardiovascular Interventions | 2 |
| JACC-Heart Failure | 2 |
| JAMA | 2 |
| JAMA Internal Medicine | 1 |
| JAMA Network Open | 2 |
| JAMA Oncology | 2 |
| Journal of Allergy And Clinical Immunology | 2 |
| Journal of Bone And Joint Surgery-American Volume | 2 |
| Journal of Crohn’s And Colitis | 1 |
| Journal Of Hepatology | 2 |
| Journal Of Internal Medicine | 2 |
| Journal of The American College Of Cardiology | 2 |
| Journal of The American College Of Surgeons | 2 |
| Journal of The American Society Of Nephrology | 2 |
| Journal of Thoracic And Cardiovascular Surgery | 2 |
| Journal Of Vascular Surgery | 2 |
| Kidney International | 2 |
| Lancet | 1 |
| Nephrology Dialysis Transplantation | 2 |
| Neurology | 1 |
| Neurosurgery | 2 |
| New England Journal of Medicine | 2 |
| Pediatric Critical Care Medicine | 2 |
| Preventive Medicine | 2 |
| Surgery for Obesity And Related Diseases | 2 |
| Transplantation | 2 |

**Appendix C. Coding instructions**

| **Variable** | **Instruction** |
| --- | --- |
| patient | Does the infographic describes the patient population? |
| patient_ind | Is the patient population described in sufficient detail so that it can be read independently from the study? For example, the population needed to include some demographic characteristics (e.g., mean age) |
| intervention | Does the infographic describes the intervention? |
| intervention_ind | Is the intervention described in sufficient detail so that it can be read independently from the study? The intervention and comparison needed to include some information on the intervention parameters (e.g., drug dose, frequency of treatment). |
| comparator | Does the infographic describes the comparator? (NB: if an observational study is a single-arm study, the compator should be coded "N/A") |
| comparator_ind | Is the comparator described in sufficient detail so that it can be read independently from the study? (NB: if an observational study is a single-arm study, the compator should be coded "N/A") The intervention and comparison needed to include some information on the intervention parameters (e.g., drug dose, frequency of treatment). |
| outcome_measure | Does the infographic describes the outcome(s)? |
| outcome_ind | Are the outcomes described in sufficient detail so that it can be read independently from the study? The outcome needed to be specific about the measure used (e.g., all-cause mortality). If the infographic mentions "pain", "QoL" but does not describe the scale or the range (0-10; 0-100), then code "No". |
| benefits | Does the infographic report on benefits  If benefits were measured BUT results a tretment was not effective, code "yes" as well |
| harms | Harms include adverse events  e.g. Complications, % people who died, etc |
| point_estimate | Are point estimates reported?  These could either be within-group (pre-post or final value at any given timepoint) point estimates OR a point estimate between groups (a between-group mean diff) |
| 95%ci | Are 95% CI reported? |
| between_group | Are between-group differences reported?  Code "yes" if either a between-group mean difference (or similar) is reported; or there are any indications of difference between groups (eg a P-value; a "*" indicating a difference between groups) |
| mcid | Is there a mention to a "clinically important effect" (or similar)? Does the infographic describe a known MCID threshold (e.g. 2/10 points for pain in back pain) |
| dichotomous_outcomes | For dichotomous outcomes, did the infographic present a clear summary statistic?  Examples include proportions (%), relative risk (RR), number needed to treat (NNT), or charts commonly used to communicate risk in absolute terms (eg Icon array) |
| bias | Whether the infographic acknowledged risk of bias, the certainty of evidence (if applicable), and study limitations. |
| Certainty | FOR REVIEWS ONLY - Did the infographic use terminology similar to the one used by GRADE?  e.g. High, moderate, low, very low certainty evidence |
| limitations | Did the infographic mention study limitations  e.g. Observational studies are not good for estimating treatment effects |
| conclusion | Did the infographic include a conclusion? |
| indirectness | Has the conclusion been based on the correct populations, interventions or outcomes (i.e. no issue with indirectness) |
| conclusion_weighed | Are conclusions weighted in relation to risk of bias/certainty of evidence? |
| conclusions_primary | Are conclusions in the infographic based on the findings from the study's primary outcome? |
| coi | Did the infographic report any conflict of interest? |
